# The Performance and Mechanism of Sludge Reduction by the Bioaugmentation Approach

**DOI:** 10.3390/life12101649

**Published:** 2022-10-20

**Authors:** Jiangwei Li, Xiaoyong Yang, Anyi Hu, Yan Li, Yeyun Li, Lijun Fu, Chang-Ping Yu

**Affiliations:** 1 CAS Key Laboratory of Urban Pollutant Conversion, Fujian Key Laboratory of Watershed Ecology, Institute of Urban Environment, Chinese Academy of Sciences, Xiamen 361021, China; 2 School of Environmental and Material Engineering, Yantai University, 30 Qingquan Road, Yantai 264005, China; 3 School of Ecological Environment and Urban Construction, Fujian University of Technology, Fuzhou 350118, China; 4 School of Petrochemical Engineering, Lanzhou University of Technology, Lanzhou 730050, China; 5 Fujian Provincial Key Laboratory of Ecology-Toxicological Effects & Control for Emerging Contaminants, Putian 351100, China; 6 Water Innovation, Low Carbon and Environmental Sustainability Research Center, National Taiwan University, Taipei 106, Taiwan

**Keywords:** excess sludge, sludge reduction, cryptic growth, optimization, response surface methodology

## Abstract

**Simple Summary:**

Activated sludge-based wastewater treatment process is one of the most popular adopted systems in wastewater treatment plants around the world. Excess sludge is an inevitable byproduct of the process, and the enormous quantity has brought a significant burden on operational costs. Various physicochemical and biological methods have been developed. Biological-based methods are promising because of less chemical consumption and low operation cost comparing to physicochemical methods. Hence, the present study is aimed at searching for functional bacteria that could reduce sludge, enhance the performance of sludge reduction through optimization, and try to unveil the underlying mechanism during sludge reduction. A total of 19 strains that belong to Firmicutes, Proteobacteria, and Actinobacteria were successfully isolated and identified. Subsequently, the performance of sludge reduction by pure culture or mix-cultures was validated. In total, 21.2% and 13.9% of total suspended and volatile suspended solids were reduced within 48 h after optimization via response surface methodology. The three-dimensional excitation-emission matrix fluorescence spectrum and hydrolases test results revealed that the sludge reduction might be promoted by the strain mainly through hydrolysis via proteinase and amylase. The results obtained from the study could help us to find an effective and economical way to resolve the sludge issue.

**Abstract:**

Millions of wastewater treatment plants (WWTPs) based on the activated sludge process have been established worldwide to help to purify wastewater. However, a vast amount of sludge is inevitably generated, and the cost of sludge disposal could reach over half of the total operation cost of a WWTP. Various sludge reduction techniques have been developed, including physicochemical, biological, and combinational methods. Micro-organisms that could reduce sludge by cryptic growth are vital to the biological approach. Currently, only limited functional bacteria have been isolated, and the lack of knowledge on the underlying mechanism hinders the technique development. Therefore, the present study is aimed at isolating sludge-reducing bacteria and optimizing the sludge reduction process through response surface methodology. Nineteen strains were obtained from sludge. The mix-cultures did not show a higher sludge reduction rate than the pure culture, which may be ascribed to the complicated interactions, such as competition and antagonistic effects. In total, 21.2% and 13.9% of total suspended and volatile suspended solids were reduced within 48 h after optimization. The three-dimensional excitation-emission matrix fluorescence spectrum and hydrolases test results revealed that the sludge reduction might be promoted by the strain mainly through hydrolysis via proteinase and amylase. The results obtained from the study demonstrate the potential of using micro-organisms for sludge reduction through cryptic growth.

## 1. Introduction

The activated sludge process is regarded as one of the most influential environmental engineering systems in the twentieth century because of its effective removal of macronutrients (e.g., carbon, nitrogen, and phosphorus) and circumvention of water ecosystem deterioration [1,2]. Consequently, millions of wastewater treatment plants (WWTPs) based on the activated sludge process have been established worldwide [3]. Despite the positive effects on sewage purification, vast amounts of excess sludge are concurrently produced in WWTPs, which leads to concentrated pollutants in the sludge. Therefore, sludge not only contains organic matters, but also contains heavy metals, pathogens, and persistent organic pollutants to cause secondary environment pollution, bringing about many environmental and economic concerns regarding sludge disposal [4,5]. For example, 6.25 million tons of dry solids were generated in China in 2013, and the number keeps increasing [3]. It was estimated that more than half of the operational budget for WWTPs was spent on sludge treatment and disposal [6].

The sludge is a complex aggregate that consists of organisms (e.g., bacteria, fungi, protozoa, and some invertebrates), organic substrates (e.g., adsorbed recalcitrant pollutants, and microbial metabolites), inorganic constituents (e.g., sands and metal elements), and water. Water content is one of the most significant factors affecting the cost of sludge transport and handling, whether via landfilling, incineration, or reuse [5]. However, sludge is difficult to dewater since it could form a water-rich networked gel. The conventional mechanical dewatering, even with flocculants, could only remove 20~30 wt% of water from the settled sludge [7]. Hence, many efforts have been made to develop innovative dewatering technologies to reduce expense and the adverse environmental impacts of sludge disposal.

Physicochemical approaches have been tested for reducing sludge. For instance, Sun et al. demonstrated that sludge solubilization and dewatering could be enhanced by freezing with the addition of nitrite. The soluble organic compounds (mainly in lower molecular weight) increased 65% after treatment [8]. The combination of citrate acid addition and microwave treatment was validated to help to interrupt the flocculated structure of sludge and decouple the extracellular polymeric substances (EPS), so that sludge solubilization was promoted [9]. Ultrasonic treatment could enhance volatile solids (VS) removal from sludge and accelerate the sludge degradation rate [10]. Other treatment methods (e.g., heating, lysis-thickening centrifuge, stirred ball mill, focused pulsed pretreatment, ozonation, and addition of alkaline) could also improve the sludge dewatering and reduction [11]. Furthermore, biological treatment is another promising way of sludge reduction. Compared with the physicochemical approaches, biological treatment shows some advantages, including lower operating and investment costs, higher sludge dewaterability, and the avoidance of caustic chemicals [11].

Among the biological sludge reduction technologies, cryptic growth is a promising way for its eco-friendliness. Cryptic growth refers to some autochthonous microbes in sludge that could utilize the organic substrates contained in the sludge or uptake other micro-organisms through aerobic respiration and help decouple the networked floc [12]. It was reported that over 40% of sludge was reduced through cryptic growth with high-pressure homogenization treatment, and the cost was estimated at only 0.177 US dollars per kilogram of dry sludge [13]. Similarly, 56% of sludge was reduced in a pilot-scale lysis-cryptic growth system, and the operation cost was 0.186 US dollars/m^3^ of wastewater [14]. However, as the most critical part during cryptic growth, the functional microbes are mostly unclear, which significantly restrains the optimization and the application of the technology. Few functional bacteria were identified, and the way they disrupt the sludge is still ambiguous.

Hence, in the present study, we first enriched and isolated some functional bacteria which could show the cryptic growth during sludge incubation. The bacteria were then identified and the performance on sludge reduction in single or mixed conditions was verified. Response surface methodology was adopted to further optimize the conditions of sludge reduction. At last, the mechanism of how the bacteria reduce sludge was investigated.

## 2. Materials and Methods

### 2.1. Sampling and Enriching

Excess sludge used for enrichment was collected from a domestic WWTP in Jimei district, Xiamen, China. The WWTP employs the activated sludge process and has a 90,000 m^3^/day capacity. 200 mL of excess sludge were incubated in 500 mL flasks with different initial pH (3, 7, and 10) at 30 °C or 50 °C (labeled as UT), 180 rpm. After one week, 100 mL of the sludge was discarded and replaced with the same amount of fresh sludge. The native bacteria that could reduce sludge were enriched by repeating the above procedure eight times.

### 2.2. Isolation and Identification

The above-mentioned enrichment was diluted with sterile water until 10^−12^. The dilutions in the range of 10^−6^~10^−12^ were spread on screening plates (15% excess sludge and 2% agar, sterilized at 121 °C for 15 min) and then placed in the incubator at 35 °C. Single colonies with transparent rings on the plates were selected and transferred to Luria-Bertani (LB) medium [15]. After purification, the strains were used for further experiments and saved in the LB medium with glycerin preservation under −80 °C.

Colony PCR of 16S rRNA gene was performed to identify the isolates. The master mix for PCR amplification was prepared prior to carrying out PCR under the following conditions: ExTaq polymerase (12.5 μL), forward primer 27F (0.5 μL), reverse primer 1492R (0.5 μL) [16], and nucleic acid-free water (12.5 μL). An appropriate amount of a bacterial colony was removed directly from the plate and placed into a PCR tube with master mix. The thermocycler program was as follows: initiated from a predenaturation at 94 °C for 4 min, followed by 30 cycles of amplification which consisted of 94 °C for 1 min, 55 °C for 1 min, and 72 °C for 2 min, and a final extension at 72 °C for 7 min. The PCR products were purified using MinElute PCR purification kit (Qiagen, Hilden, Germany) and sent to Meiji Biomedicine Technology Co., Ltd. (Shanghai, China) for sequencing. The sequences were submitted to NCBI and identified using the EzBio Cloud database (https://www.ezbiocloud.net/identify, accessed on 12 July 2022) and the reference sequences of type species were retrieved from the GenBank database. Phylogenetic analysis was performed using MEGA version X [17] after multiple alignments of data by CLUSTAL W function [18] with default parameters. Distances (distance options according to the Kimura two-parameter model) and clustering with the neighbor-joining method [19] were determined by using bootstrap values based on 1000 replications.

### 2.3. Sludge Reduction Experiments

A total of 50 mL of excess sludge (with initial total suspended solids (TSS) of approximately 1500 mg/L, volatile suspended solids (VSS) of approximately 1000 mg/L, and soluble chemical oxygen demand (SCOD) of approximately 300 mg/L) were taken for reduction experiments, and triplicates were conducted in all experiments. The candidate strain was inoculated in a 50 mL disposable sterile tube which contained 20 mL LB liquid medium and cultivated for 24 h at 180 rpm, 35 °C. Then, the culture solution was centrifuged at 14,000 rpm for 1 min, the supernatant was discharged, washed with sterile normal saline 3 times, and diluted into the working concentration (with the exact number of approximately 10^8^ CFU/mL) with sterile normal saline. Mixed cultures were prepared by combining the candidate strains in equal proportions. A total of 10 mL pure culture or mix-cultures as the inoculation were added into the above sludge. After incubation, the performance of sludge reduction was validated by comparing the concentration of SCOD, TSS, and VSS. Afterwards, the response surface methodology (RSM) was applied to optimize the conditions (temperature, pH, and incubation time) of sludge reduction, the Box–Behnken method was used to design the response surface experiment [20]. At last, the dynamic concentration of protease and amylase, and the composition of sludge during the incubation process were measured to explore the reduction mechanism of the excess sludge by bioaugmentation approach.

### 2.4. Physicochemical Analysis

SYBR Green-based flow cytometry was used to quantify the strain number [21], and the fluorescence intensity was quantified by a flow cytometer (FlowSight, Merck Millipore). To determine SCOD, the sludge was first centrifuged at 3000 g/min for 10 min and then the supernatant was filtered by a membrane (0.45 μm pore size). The SCOD in the supernatant was measured using a commercial COD kit (Lian-Hua Tech Co., Ltd., Beijing, China). An adequate amount (V_0_ L) of sludge was taken from a flask and put on a cleaned glass fiber filter. After dewatering by Nutsch filter, the sludge was transferred to an oven set at 105 °C. The TSS (g/L) is the value of the weight (W_1_ g) of the dried sludge divided by the volume (V_0_ L). The sludge was further ashed at 550 °C for 15 min in a Muffle furnace. After cooling, the weight of sludge (W_2_ g) was used to calculate the VSS ((W1 − W2)/V_0_, g/L). Total phosphorus (TP) and total nitrogen (TN) were monitored according to the standard method (APHA, 2005). The contents of protease and amylase were determined as in a previous study [22]. Three-dimensional excitation-emission matrix (3D-EEM, F-4600 spectrofluorimeter, Hitachi, Japan) fluorescence spectroscopy was utilized to monitor the dynamic change of the sludge composition during the reduction process [23]. For the statistical analysis, a paired t test was used to determine differences between the treatments using the software OriginPro 8.0 (OriginLab Corporation, Northampton, MA, USA). The significance level was assumed to be 95%.

## 3. Results and Discussion

### 3.1. Isolation and Identification of Sludge-Reductants

Nineteen strains were eventually isolated and purified from enrichment culture. The strains are listed in Table 1. Among these strains, seven were isolated from initial pH of 3; three were obtained from a neutral pH of 7; eight were isolated from an alkaline pH of 10; and one was obtained from the enrichment without pH adjustment and under a high-temperature condition (50 °C). Based on sequencing and BLAST, these strains were assigned to three phyla: five belonged to Actinobacteria, seven belonged to Firmicutes, and the other seven strains were classified into Proteobacteria. The initial pH showed substantial impacts on the enrichment. Specifically, no Firmicutes were isolated from an acidic pH condition, and no Actinobacteria could be enriched under alkaline pH conditions. Comparatively, the neutral pH conditions showed no selectivity. The dominant populations of Firmicutes and Proteobacteria were also mostly investigated in activated sludge [24,25]. Therefore, the fact that more functional bacteria were isolated from these two phyla implied that there is a potential storehouse for more versatile bacteria. Acid and alkaline treatment can accelerate sludge decomposition, and the released organic substrates can thus be accessible [26,27]. The ability of growth at acidic (pH = 3) or alkaline (pH = 10) conditions of the isolates indicated that acid/alkaline pretreatment could be combined with the cryptic biological growth; thereby, a higher performance could be expected.

Strain WAS-3-5-1, WAS-UT-2, and WAS-3-10-2, were selected for the following experiments based on the diameter of the transparent ring, while culturing on sludge plates. Strain WAS-3-5-1 and WAS-UT-2 shared the highest sequence similarity with *Humibacter ginsengiterrae* and *Bacillus anthracis*, respectively, whose taxonomical status were also confirmed by their phylogenetic tree (Figures S1 and S2). *Bacillus* spp. are well known for their strong adaptability to a harsh environment, whose capacity of sludge reduction also have been reported in previous studies [28,29]. Although strain WAS-3-10-2 showed the highest sequence similarity to *Novosphingobium tardum* CC-ALB-2^T^, the distinct phylogenetic relationships between strain WAS-3-10-2 and other genera in the family of *Sphingomonadaceae* (Figure S3) revealed that strain WAS-3-10-2 cannot be attributed to any genus, which should be considered as a novel member of the family *Sphingomonadaceae*.

**Table 1:** The BLAST results of the isolates.

Name	Accession No.	Top-Hit Strain	Top-Hit Phylum	Similarity (%)	Completeness (%)
WAS-3 ^a^-5-1	OP379642	*Humibacter ginsengiterrae* DCY60(T)	Actinobacteria	93.1	98.1
WAS-3 ^a^-5-2	OP379643	*Xinfangfangia soli* ZQBW(T)	Proteobacteria	97.4	100
WAS-3 ^a^-10-1	OP379637	*Flexivirga lutea* TBS-100(T)	Actinobacteria	91.8	95.6
WAS-3 ^a^-10-2	OP379638	*Novosphingobium arabidopsis* CC-ALB-2(T)	Proteobacteria	94.4	100
WAS-3 ^a^-10-3	OP379639	*Microbacterium flavum* YM18-098(T)	Actinobacteria	94.9	99.0
WAS-3 ^a^-11-1	OP379640	*Kocuria salina* Hv14b(T)	Actinobacteria	95.2	96.8
WAS-3 ^a^-12-1	OP379641	*Noviherbaspirillum soli* SUEMI10(T)	Proteobacteria	90.7	100
WAS-7 ^a^-8-1	OP379646	*Rhodanobacter terrae* GP18-1(T)	Proteobacteria	98.6	100
WAS-7 ^a^-10-1	OP379644	*Staphylococcus warneri* ATCC 27836(T)	Firmicutes	91.9	99.7
WAS-7 ^a^-10-3	OP379645	*Cellulomonas massiliensis* JC225(T)	Actinobacteria	92.3	100
WAS-10 ^a^-6-1	OP379632	*Diaphorobacter oryzae* RF3(T)	Proteobacteria	95.1	96.6
WAS-10 ^a^-6-2	OP379633	*Bacillus aryabhattai* B8W22(T)	Firmicutes	94.0	100
WAS-10 ^a^-9-1	OP379634	*Bacillus sporothermodurans* M215(T)	Firmicutes	88.9	99.5
WAS-10 ^a^-9-2	OP379635	*Bacillus vini* LAM0415(T)	Firmicutes	88.7	99.1
WAS-10 ^a^-9-3	OP379636	*Bacillus cereus* ATCC 14579(T)	Firmicutes	99.2	100
WAS-10 ^a^-10-1	OP379629	*Luteimonas cucumeris* CGMCC 1.10821(T)	Proteobacteria	93.1	100
WAS-10 ^a^-12-1	OP379630	*Bacillus oryzaecorticis* R1(T)	Firmicutes	96.3	75.6
WAS-10 ^a^-12-2	OP379631	*Ottowia pentelensis* RB3-7(T)	Proteobacteria	92.1	100
WAS-UT ^b^-2	OP379647	*Bacillus anthracis* Ames (T)	Firmicutes	99.2	100

^a^ The number (3, 7, and 10) after WAS indicates the initial pH during enrichment. ^b^ UT indicates the strain was isolated from the sludge without pH adjustment and under high-temperature conditions (50 °C).

### 3.2. Sludge Reduction by Pure or Mixed Cultures

**Figure 1:**
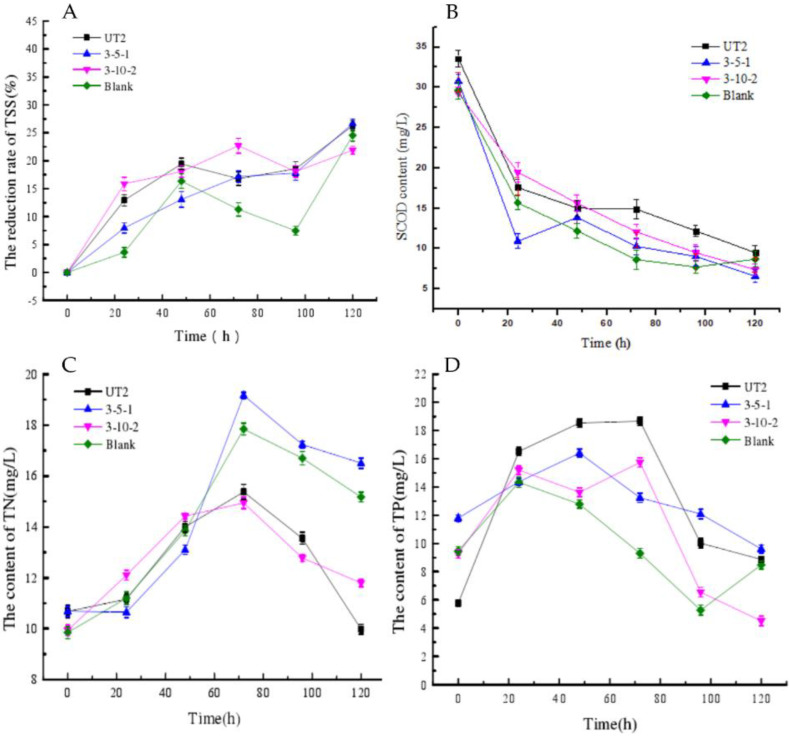
The concentration variation of TSS (**A**), SCOD (**B**), TN (**C**), and TP (**D**) during the sludge reduction process by pure cultures (strain WAS-UT2, WAS-3-5-1 and WAS-3-10-2).

Sludge reduction by the three selected strains was further validated in flasks at 35 °C, 180 rpm. Though similar removal of TSS (21.9~26.7%) were obtained after 120 h (Table S1), it is clear that the sludge reduction process has been significantly promoted by the isolated strains in the first 24 h (Figure 1A). In the blank control test, only less than 3% of TSS was reduced. Comparatively, the highest TSS reduction of over 15.8% was obtained by WAS-3-10-2, followed by WAS-UT-2 (12.5%) and WAS-3-5-1 (7.8%). The SCOD gradually decreased during incubation (Figure 1B). More than half of the SCOD was removed in the first 48 h, and WAS-3-5-1 showed the highest removal rate (63%) in the first 24 h. The contents of TN and TP were also monitored during experiments (Figure 1C,D). During sludge reduction, TN and TP increased firstly and then decreased. The highest TN concentrations (14.9~19.2 mg/L) were observed at 72 h, while the highest TP concentrations (14.3~18.7 mg/L) were observed during 24~72 h. The TN concentration almost doubled (1.8 times) in 72 h in the sludge incubated with WAS-3-5-1. Total SCOD with the volatile fatty acids as the predominant fraction is an important factor to its availability in nitrogen and phosphorus removal [30]. The decreasing of SCOD in this study may enhance the biological phosphorus removal process [31]. From the variation trend of sludge properties during the whole reduction process (Table S1) by pure culture, there were no obvious differences between the pure culture of WAS-3-10-2 and the control group (*p* > 0.05). While the concentration of SCOD and the reduction rate of TSS in the culture of WAS-UT2 showed significant difference from the control group (*p* < 0.01 and 0.05, respectively), the culture of WAS-3-5-1 exhibited significant difference with the control group in the content of TP (*p* < 0.05) (Table S2).

**Figure 2:**
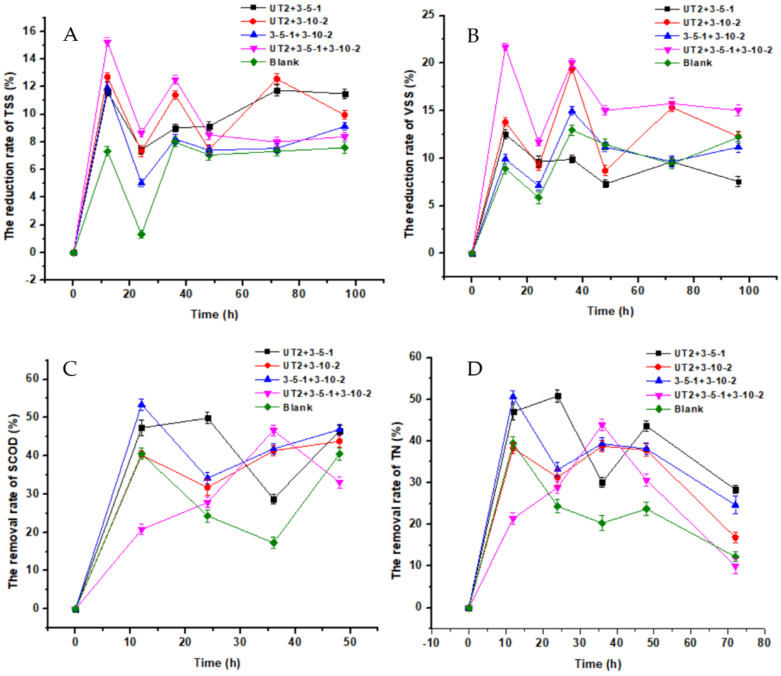
The concentration variation of TSS (**A**), VSS (**B**), SCOD (**C**), and TN (**D**) during the sludge reduction process by mix-cultures (strain WAS-UT2+WAS-3-5-1, strain- WAS-UT2+ WAS-3-10-2, strain WAS-3-5-1+ WAS-3-10-2 and WAS-UT2+WAS-3-5-1+ WAS-3-10-2).

After the determination of sludge reduction by pure culture, the reduction by mix-cultures was also examined at the same condition (35 °C, 180 rpm) to verify the possible synergistic effects (Figure 2). Higher TSS reduction rates (8~12%) were achieved after 96 h by mix-cultures compared to the blank control (7%); Approximately 12% of TSS was reduced after 96 h by WAS-UT-2 and WAS-3-5-1, while the highest TSS reduction rate of 15.2% was obtained by the mixture of the three strains at 12 h (Table S3). Nevertheless, the TSS reduction rates by the mix-cultures were less than the pure culture (Figure 2A). Similarly, the most VSS reduction of 22% was achieved after 12 h when cultivated with WAS-UT-2, WAS-3-5-1, and WAS-3-10-2. Differently, the VSS reduction by WAS-UT-2 and WAS-3-5-1 was lower than the blank control (Figure 2B). In terms of organics, the SCOD contents quickly declined in the first 12 h and afterwards fluctuated along reaction time, which indicated a complex process. After 48 h, 45.2% of SCOD were removed by WAS-UT-2 and WAS-3-5-1, while less SCOD (33%) was removed by the three strains, which was lower than the control (Figure 2C). The nitrogen removal showed a distinct trend from SCOD during sludge reduction. Firstly, 20~50% of nitrogen was quickly removed in the first 12 h, and the WAS-3-10-2 and WAS-3-5-1 manifested the highest removal rate. Comparatively, the three mixed strains showed the poorest nitrogen removal at 12 h and 72 h but the highest removal at 36 h (Figure 2D), which implied a complex interspecies interaction.

Compared with the results from pure culture incubation after 72 h, the reduction rate of TSS was significantly (*p* < 0.05) decreased by the mix-cultures (Table S4). The results indicated complex interactions between different strains and the native microbes in the sludge. Besides the synergetic effects, the organisms may have competitive, antagonistic, or predatory relationships [32]. Therefore, pure culture addition was adopted in the subsequent experiments. Most organic removal was found in the first 12~24 h, which was consistent with the TSS removal, implying that there were many refractory substrates in the sludge, and the dissolution of biodegradable substrates is the speed-limiting factor in the process of sludge reduction. Hence, multiple pretreatment methods that can promote the lysis of sludge may benefit the bacterial reduction of sludge.

### 3.3. Optimization of Sludge Reduction

Temperature, pH, and incubation time are the three crucial factors affecting the performance of sludge reduction [33]. Response surface methodology was employed to optimize the abovementioned operational parameters using the Box–Behnken model. A total of 17 experiments were designed and conducted, as shown in Table 2. The results were fitted using a multiple quadratic regression model, and an equation was obtained where Y represents the TSS removal, X_1_, X_2_, and X_3_ represent temperature, pH, and incubation time, respectively. The equation was evaluated by the analysis of variance (ANOVA) (Table 3). The *p*-value (0.0016) was far less than 0.005, indicating that the obtained model is significant and could be used to estimate the effect of each parameter. Temperature (X_1_, *p* = 0.0011) and incubation time (X_3_, *p* = 0.02) are more important than pH (X_2_, *p* = 0.2034) for TSS removal because of the lower *p*-value.

$$\begin{array}{c} Y=26.87742-0.27662X_{1}-1.60814X_{2}-0.57460X_{3}+0.027035X_{1}X_{2}+0.012890X_{1}X_{3}\\ \;\;\;\;+0.012658X_{2}X_{3} \end{array}$$

**Figure 3:**
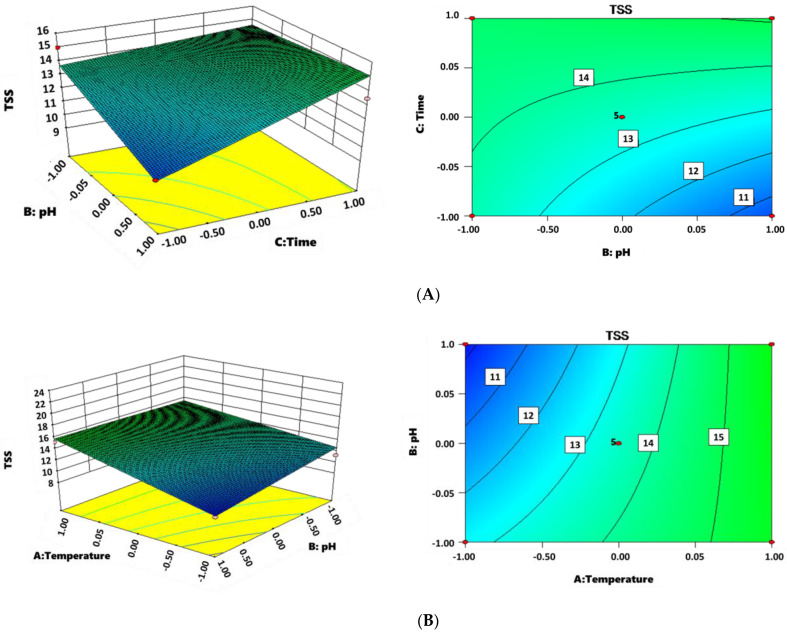

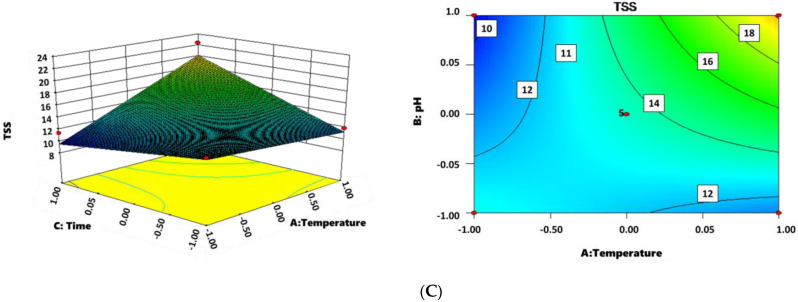
TSS variations against pH and Time (**A**); TSS variations against Temperature and pH (**B**); TSS variations against Time and Temperature (**C**).

Response surface plots were displayed in Figure 3. All three parameters showed a positive relationship with TSS removal in the tested range. Though higher temperature resulted in a higher TSS removal, it also meant the higher energy cost. The impact of temperature (in the range of 50~80 °C) on sludge pretreatment was investigated by Uma Rani et al. and showed that optimal sludge solubilization was achieved at 60 °C [34]. It was speculated that more refractory byproducts might be generated at higher temperature conditions and inhibited the sludge reduction [34]. Alkaline pretreatment is a commonly used method for sludge dewatering because it can transfer pellet and tightly bound EPS (T-EPS) fractions to the slime and loosely bound EPS (L-EPS) and therefore make the organics more biodegradable [35]. It was found that the higher alkali dose (pH 12) liberated more proteins and carbohydrates from the inner fractions to outer fractions in the sludge; however, the most increased biogas production during anaerobic digestion was obtained from the sludge pretreated at pH 10, which was assumed that the micro-organism might be inhibited at higher pH condition [35]. The sludge reduction was then validated at the optimal operational condition (50 °C, pH 10, 48 h). In total, 21.2% and 13.9% of TSS and VSS were reduced, respectively.

### 3.4. The Study of the Sludge Reduction Mechanism

**Figure 4:**
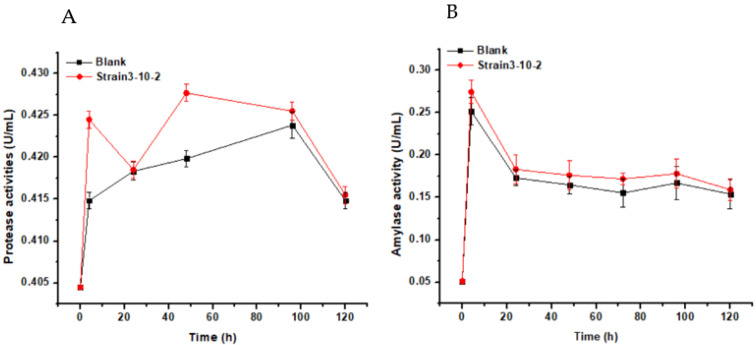
The variation of the protease activity (**A**) and the amylase activity (**B**) during the sludge reduction process by strain WAS-3-10-2.

**Figure 5:**
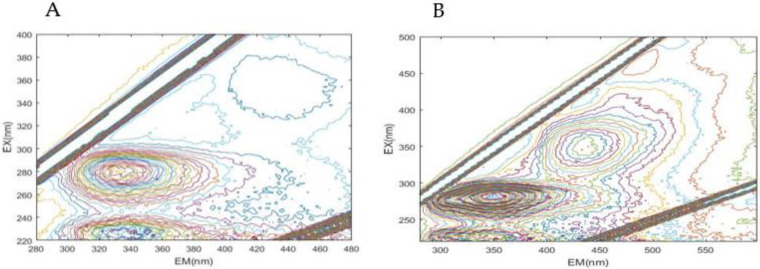
The EEM spectrum for proteins (Peak a and c), fulvic acid-like substances (Peak b and d), and humic acid-like constituents (Peak e) of the sludge before (**A**) and after (**B**) treatment (Peak a: EX/EM = 225–240/340–350 nm; Peak b: EX/EM = 240–270/370–440 nm; Peak c: EX/EM = 260–290/300–350 nm; Peak d: EX/EM = 310–360/370–450 nm; Peak e: EX/EM = 350–440/430–510 nm).

The mechanism of sludge reduction was investigated. The lysis is regarded as the time-limiting step during sludge solubilization [36], and hydrolases (e.g., protease and amylase) secreted by functional bacteria could promote the process [37,38]. More proteases were detected after the addition of WAS-3-10-2 compared with the blank set, especially at 12 (0.425 U/mL) and 48 h (0.428 U/mL) (Figure 4A). Comparatively, the amylase was always slightly higher than the blank during incubation (Figure 4B). The results herein implied that the strain contributed to sludge solubilization mainly by hydrolyzing the protein fractions in the sludge. The changes in organic fractions, including proteins, fulvic acid-like substances, and humic acid-like constituents, were revealed by 3D-EEM spectra (Figure 5). It can be seen that the signal response of all three kinds of materials significantly increased after the proposed treatment. The EPS consisting of polysaccharides, proteins, and lipids may account for over 80% of the sludge and is the main hindrance in sludge dewatering [23,39]. Therefore, it can be assumed that the strain WAS-3-10-2 could secrete both protease and amylase to help dissolve the sludge and consume the biodegradable substances. The change revealed by 3D-EEM further verified the disintegration of the sludge and the release of byproducts during the treatment process.

## 4. Conclusions

In this study, nineteen strains belonging to Firmicutes, Proteobacteria, and Actinobacteria were isolated and identified for excess sludge reduction. The pure culture rather than mixed cultures showed a higher sludge reduction rate. Bioaugmentation could be a promising approach to promote sludge reduction, which may enhance the hydrolysis yield by secreting more proteinase and amylase. After optimization, sludge reduction by strain WAS-3-10-2 could achieve the TSS and VSS removal rates of 21.2% and 13.9% under the conditions of 50 °C, pH 10, 48 h.

## Figures and Tables

**Table 2:** The set of response surface experiments based on the Box–Behnken model and the corresponding results.

No.	X_1_ ^a^ (Temperature)	X_2_ ^b^ (pH)	X_3_ ^c^ (Incubation Time)	TSS Removal (%)
1	0	0	0	13.44
2	−1	0	−1	22.41
3	−1	0	1	11.87
4	0	−1	−1	13.51
5	1	0	−1	11.40
6	0	−1	1	10.60
7	0	0	0	13.44
8	0	1	−1	14.24
9	1	0	1	13.24
10	1	−1	0	9.53
11	−1	1	0	13.71
12	0	0	0	13.44
13	1	1	0	11.27
14	0	0	0	11.27
15	0	0	0	11.27
16	−1	−1	0	15.21
17	0	1	1	14.98

^a^ The level of −1, 0, and 1 refers to 30 °C, 40 °C, and 50 °C, respectively. ^b^ The level of −1, 0, and 1 refers to pH 4, pH 7, and pH 10, respectively. ^c^ The level of −1, 0, and 1 refers to 12 h, 24 h, and 48 h, respectively.

**Table 3:** The ANOVA test of the obtained model.

Source of Variation	Sum of Squares	Degree	of Mean	F-Value	Prob > F	Significance
Equation	102.01	6	17.00	8.80	0.0016	significant
X_1_	39.42	1	39.42	20.4	0.0011	—
X_2_	3.58	1	3.58	1.85	0.2034	—
X_3_	14.77	1	14.77	7.64	0.0200	—
X_1_X_2_	2.63	1	2.63	1.36	0.2704	—
X_1_X_3_	38.28	1	38.28	19.81	0.0012	—
X_2_X_3_	3.32	1	3.32	1.72	0.2191	—
Residual	19.33	10	1.93	—	—	—
Lack of fit analysis	19.33	6	3.22	—	0.1819	not significant
Pure error	0	4	0.000	—	—	—
Sum	121.33	16	—	—	—	—

## Data Availability

Not applicable.

## Supplementary Materials

The following are available online at https://www.mdpi.com/article/10.3390/life12101649/s1, Figure S1: The neighbor-joining phylogenetic tree of WAS-3-5-1 and reference strains; Figure S2: The neighbor-joining phylogenetic tree of WAS-UT2 and reference strains; Figure S3: The neighbor-joining phylogenetic tree of WAS-3-10-2 and reference strains. Table S1: Variation of sludge properties during the reduction process by pure culture; Table S2: Results of Paired Samples Test between pure culture at the 0.05 level; Table S3: Variation of sludge properties during the reduction process by mixed culture; Table S4: Results of Paired Samples Test between mixed cultures at the 0.05 level.
