# Supplementary material for: The Performance and Mechanism of Sludge Reduction by the Bioaugmentation Approach
Source: Life (Basel). 2022 Oct 20;12(10):1649. doi: 10.3390/life12101649 (PMC9605661; doi:10.3390/life12101649)
Supplement: Supplementary file 1 [file life-12-01649-s001.zip › life-1934593-supplementary.pdf]

life

MDPI

Supplemental Materials

The Performance and Mechanism of Sludge Reduction by the Bioaugmentation Approach

Jiangwei Li [1†], Xiaoyong Yang [2†], Anyi Hu [1], Yan Li [3], Yeyun Li [1,5], Lijun Fu[4] and Chang-Ping Yu[1,6,*]

[1] CAS Key Laboratory of Urban Pollutant Conversion, Fujian Key Laboratory of Watershed Ecology, Institute of Urban Environment, Chinese Academy of Sciences, Xiamen 361021, China
[2] School of Environmental and Material Engineering, Yantai University, 30 Qingquan Road, Yantai 264005, China
[3] School of Ecological Environment and Urban Construction, Fujian University of Technology, Fuzhou 350118, China
[4] School of Petrochemical Engineering, Fujian Provincial Key Laboratory of Ecology-Toxicological Effects & Control for Emerging Contaminants, Putian, 351100, Fujian, China
[5] Lanzhou University of Technology, Lanzhou, 730000, China
[6] Water Innovation, Low Carbon and Environmental Sustainability Research Center, National Taiwan University, Taipei, 106, Taiwan
* Correspondence: cpyu@ntu.edu.tw
† These authors contributed equally to this work

Table S1. Variation of sludge properties during the reduction process by pure culture.

	**Blank**	**WAS-UT2**	**WAS-3-5-1**	**WAS-3-10-2**	**Blank**	**WAS-UT2**	**WAS-3-5-1**	**WAS-3-10-2**
Time(h)	Mean of Reduction rate of TSS (%)				Std. Error Mean			
24	3.63636	12.93189	7.97546	15.86345	0.9	1.02	1.1	0.87
48	16.36364	19.44719	13.08793	18.07229	1.4	1	0.8	1.3
72	11.31313	16.78184	17.17791	22.69076	1	1.2	0.89	1.2
96	7.47475	18.55874	17.79141	18.07229	1.3	1.3	1.2	0.8
120	24.54545	26.25864	26.68712	21.88755	0.8	0.9	1.3	1
	Mean of TN content (mg/L)				Std. Error Mean			
0	9.845	10.67333	10.68667	9.93333	0.2	0.24	0.12	0.23
24	11.19833	11.15833	10.65	12.1	0.21	0.2	0.11	0.25
48	13.84667	14	13.09667	14.42167	0.18	0.21	0.18	0.2
72	17.8425	15.375	19.165	14.93833	0.12	0.3	0.2	0.24
96	16.7	13.5475	17.23333	12.77833	0.14	0.23	0.23	0.27
120	15.17167	9.9775	16.505	11.79667	0.2	0.2	0.22	0.2
	Mean of TP content (mg/L)				Std. Error Mean			
0	9.46458	5.76949	11.80228	9.27605	0.24	0.24	0.32	0.32
24	14.32852	16.52379	14.36623	15.19574	0.23	0.3	0.33	0.35
48	12.78262	18.53891	16.40229	13.62469	0.28	0.31	0.38	0.3
72	9.30119	18.66459	13.23508	15.74455	0.32	0.3	0.3	0.34
96	5.27932	10.01758	12.08507	6.54244	0.34	0.34	0.33	0.37
120	8.48424	8.8613	9.61539	4.50637	0.3	0.21	0.38	0.3
	Mean of SCOD content (mg/L)				Std. Error Mean			
0	29.655	33.565	30.775	29.28833	0.8	1	1.2	1.1
24	15.685	17.58667	10.92	19.48333	0.9	1.02	1.1	0.87
48	12.20333	15.0325	13.87167	15.66167	0.8	1	0.8	0.9
72	8.63667	14.89	10.27833	12.075	1	1.2	0.89	1.2
96	7.70667	12.2075	9.03667	9.49833	1.2	0.7	1.2	0.8
120	8.71	9.495	6.59	7.395	0.8	0.9	0.9	1

Table S2. Results of Paired Samples Test between pure culture at the 0.05 level.

	Paired Samples Test (At the 0.05 level)											
	TN			TP			SCOD			Reduction rate of TSS		
Pair	t	DF	p	t	DF	p	t	DF	p	t	DF	p
Blank—WAS-3-5-1	1.22618	5	0.27472	-3.05804	5	0.02816*	0.17239	5	0.86989	-1.66975	5	0.15584
Blank—WAS-UT2	1.71942	5	0.14617	-1.68215	5	0.15337	-4.22463	5	0.00829**	-2.86876	5	0.03505*
Blank—WAS-3-10-2	1.61168	5	0.16795	-0.64127	5	0.54958	-2.02076	5	0.09927	-2.06092	5	0.09432

* significantly ($p < 0.05$);

** extremely significantly ($p < 0.01$);

Table S3. Variation of sludge properties during the reduction process by mixed culture.

	Blank	UT2+3-5-1	3-5-1+3-10-2	UT2+3-10-2	UT2+3-5-1+3-10-2	Blank	UT2+3-5-1	3-5-1+3-10-2	UT2+3-10-2	UT2+3-5-1+3-10-2
Time(h)	Mean of Reduction rate of TSS (%)					Std. Error Mean				
12	7.32	11.62	11.90	12.70	15.23	0.39	0.3	0.33	0.32	0.35
24	1.33	7.44	5.03	7.33	8.65	0.28	0.31	0.38	0.31	0.3
36	7.99	9.01	8.20	11.39	12.52	0.32	0.3	0.3	0.33	0.34
48	7.06	9.14	7.41	7.46	8.52	0.34	0.34	0.33	0.34	0.37
72	7.32	11.75	7.54	12.57	8.00	0.3	0.4	0.38	0.35	0.3
96	7.59	11.49	9.13	9.95	8.39	0.29	0.33	0.3	0.288	0.39
	Mean of Reduction rate of VSS (%)					Std. Error Mean				
12	8.90585	12.5	9.89848	13.81074	21.71838	0.49	0.5	0.44	0.39	0.53
24	5.85242	9.63542	7.1066	9.20716	11.69451	0.41	0.6	0.48	0.41	0.6
36	12.9771	9.89583	14.97462	19.43734	20.04773	0.43	0.4	0.5	0.43	0.54
48	11.45038	7.29167	11.16751	8.69565	15.0358	0.48	0.41	0.55	0.48	0.57
72	9.41476	9.63542	9.64467	15.34527	15.75179	0.56	0.43	0.45	0.61	0.5
96	12.21374	7.55208	11.16751	12.27621	15.0358	0.59	0.55	0.53	0.55	0.59
	Mean of Reduction rate of SCOD (%)					Std. Error Mean				
12	40.53532	47.30898	53.41828	40.20742	20.79566	1.45	2	1.2	1.39	1.53
24	24.32395	49.86265	34.19391	31.75764	27.83419	1.42	1.5	2.1	1.41	1.6
36	17.28753	28.74148	41.85042	41.33188	46.66852	1.43	1.22	1.4	1.43	1.54
48	40.49393	46.61715	46.89197	43.79913	33.13395	1.4	1.3	1.5	1.48	1.57
	Mean of Reduction rate of TN (%)					Std. Error Mean				
12	39.47695	46.9812	50.64776	38.27398	21.4108	1.45	2	1.2	1.39	1.53
24	24.34308	50.79537	33.15312	31.293	28.85046	1.7	1.5	2.1	1.41	1.6
36	20.31482	30.05242	39.3349	38.76973	43.87907	1.43	1.22	1.4	1.43	1.8
48	23.71096	43.58279	38.11078	37.82881	30.61707	1.4	1.3	1.5	1.48	1.57
72	12.24591	28.36226	24.65572	16.86564	10.06469	2.1	1	1.2	1.8	1.2

Table S4. Results of Paired Samples Test between mixed cultures at the 0.05 level.

	Paired Samples Test (At the 0.05 level)											
	Reduction rate of TSS			Reduction rate of VSS			Reduction rate of SCOD			Reduction rate of TN		
Pair	t	DF	p	t	DF	p	t	DF	p	t	DF	p
Blank—UT2+3-5-	-3.82878	6	0.00867**	0.46588	6	0.65774	-2.32295	4	0.08087	-3.43942	5	0.01845*
Blank—3-5-1+3-10-2	-2.13004	6	0.07721	-1.15437	6	0.29225	-2.6422	4	0.05745	-4.20943	5	0.00841**
Blank—UT2+3-10-2	-3.538	6	0.01225*	-1.93614	6	0.10098	-1.5276	4	0.20133	-2.24932	5	0.07434
Blank—UT2+3-5-1+3-10-2	-2.58025	6	0.04176*	-3.60063	6	0.01136*	-0.14298	4	0.89322	-0.44375	5	0.67577
Pure—Mixed	5.09411	2	0.03644*									

* significantly ($p < 0.05$);

* extremely significantly ($p < 0.01$);

Figure S1. The neighbor-joining phylogenetic tree of WAS-3-5-1 and reference strains (bootstrap was 1000), with *Timonella senegalensis* JC301^T as outgroup.

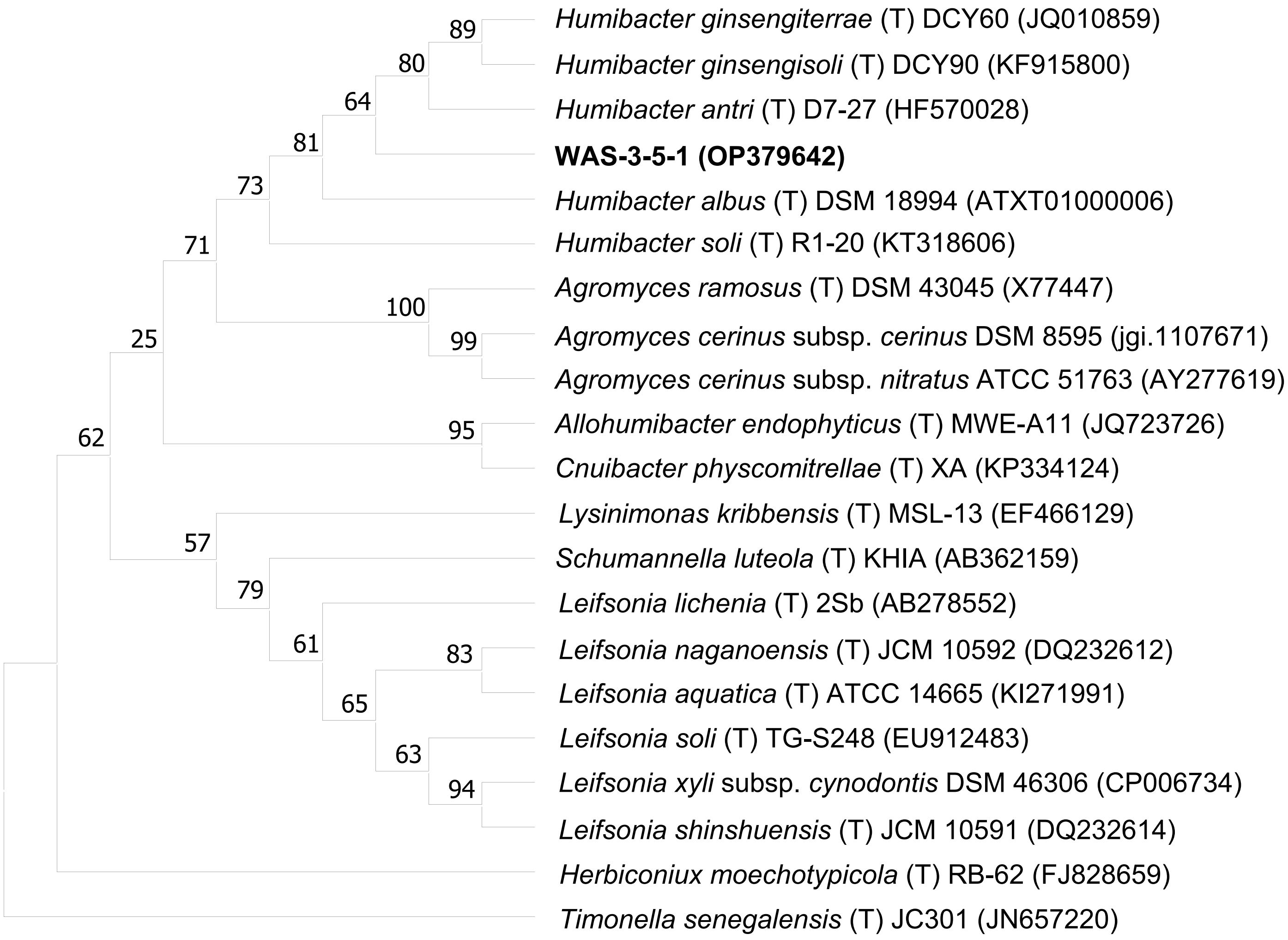

Figure S2. The neighbor-joining phylogenetic tree of WAS-UT2 and reference strains (bootstrap was 1000), with *Thermolongibacillus kozakliensis* E173a^{T} as outgroup.

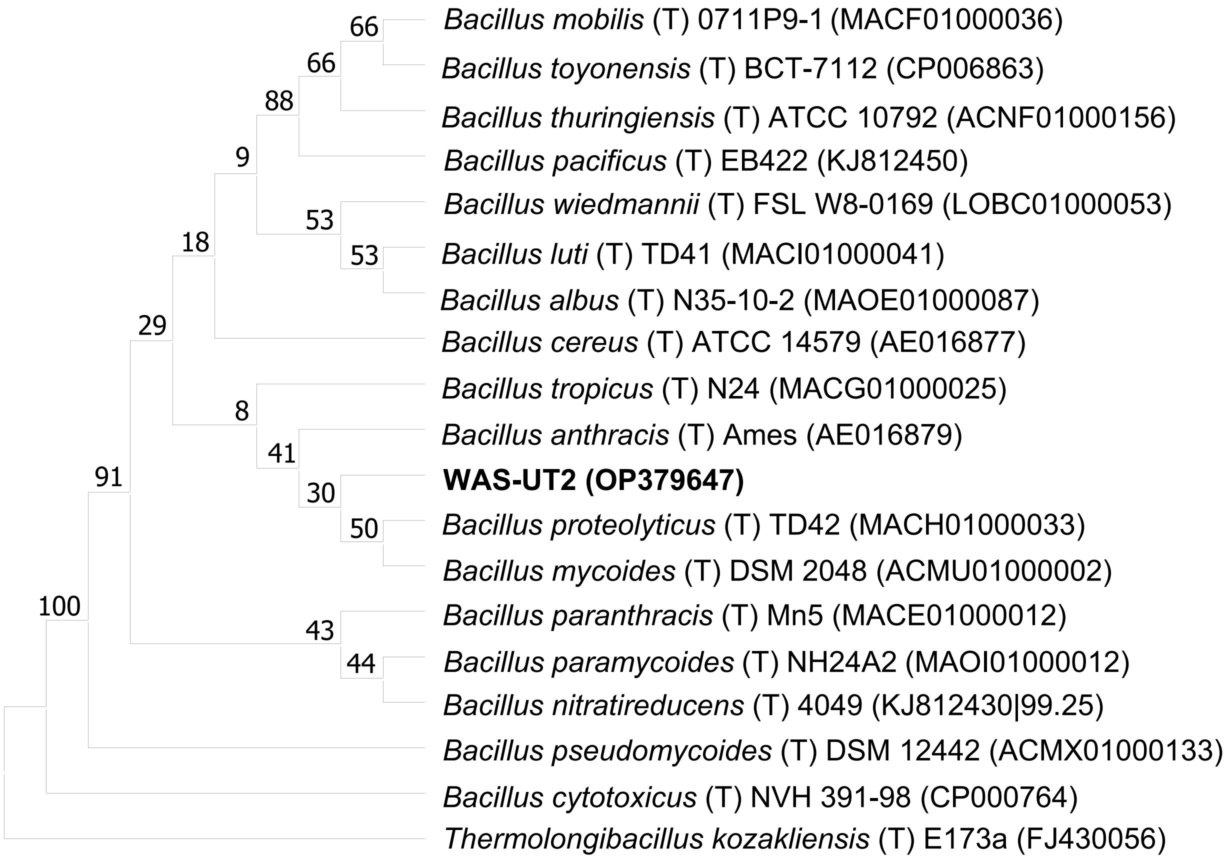

Figure S3. The neighbor-joining phylogenetic tree of WAS-3-10-2 and reference strains (bootstrap was 1000), with *Zymomonas mobilis* ATCC10988^{T} and ATCC 29192 ^{T} as outgroup.

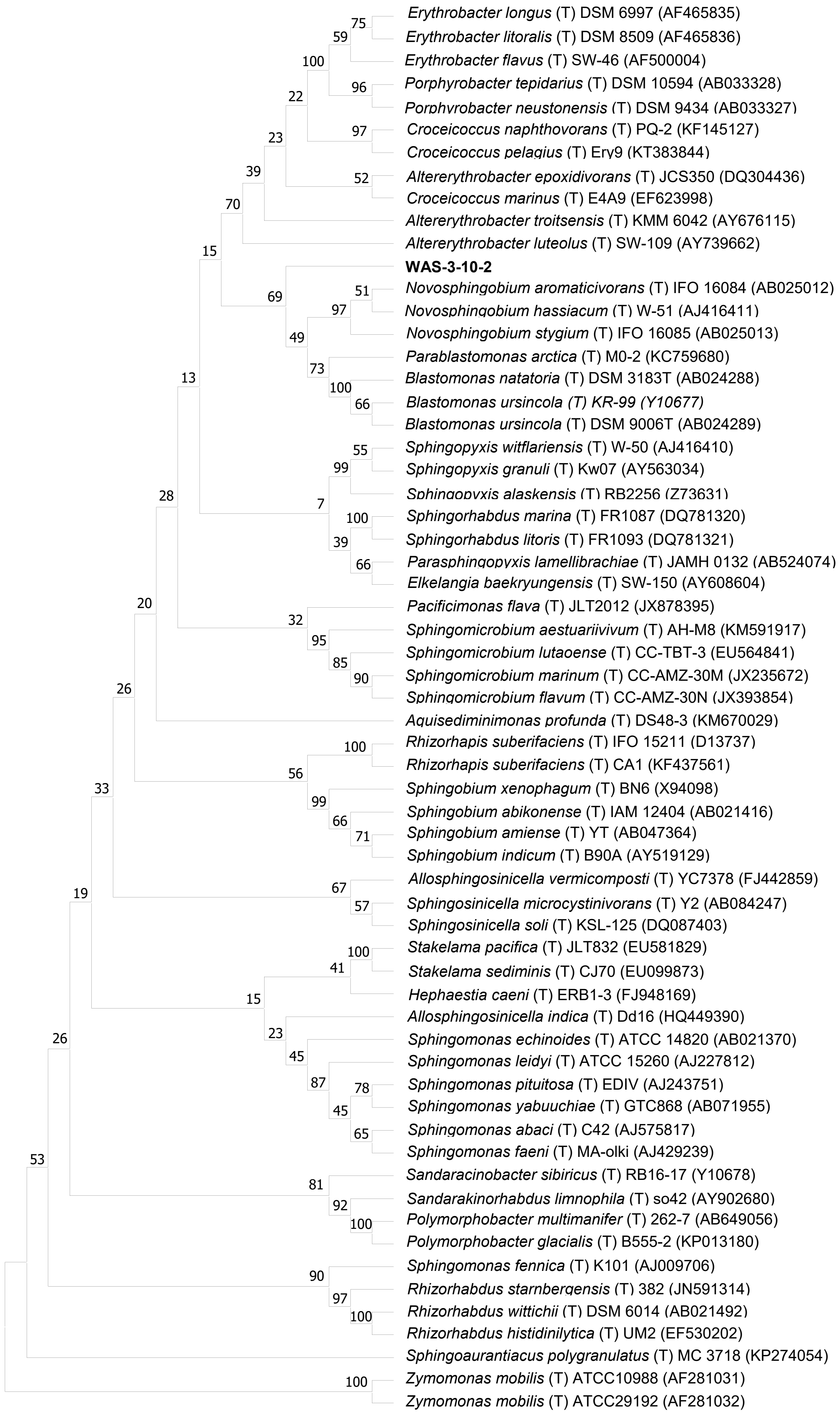
Erythrobacter longus (T) DSM 6997 (AF465835)
Erythrobacter litoralis (T) DSM 8509 (AF465836)
Erythrobacter flavus (T) SW-46 (AF500004)
Porphyrobacter tepidarius (T) DSM 10594 (AB033328)
Porphyrobacter neustonensis (T) DSM 9434 (AB033327)
Croceicoccus naphthovorans (T) PQ-2 (KF145127)
Croceicoccus pelagius (T) Ery9 (KT383844)
Altererythrobacter epoxidivorans (T) JCS350 (DQ304436)
Croceicoccus marinus (T) E4A9 (EF623998)
Altererythrobacter troitsensis (T) KMM 6042 (AY676115)
Altererythrobacter luteolus (T) SW-109 (AY739662)
WAS-3-10-2
Novosphingobium aromaticivorans (T) IFO 16084 (AB025012)
Novosphingobium hassiacum (T) W-51 (AJ416411)
Novosphingobium stygium (T) IFO 16085 (AB025013)
Parablastomonas arctica (T) M0-2 (KC759680)
Blastomonas natatoria (T) DSM 3183T (AB024288)
Blastomonas ursincola (T) KR-99 (Y10677)
Blastomonas ursincola (T) DSM 9006T (AB024289)
Sphingopyxis witflariensis (T) W-50 (AJ416410)
Sphingopyxis granuli (T) Kw07 (AY563034)
Sphingopyxis alaskensis (T) RB2256 (Z73631)
Sphingorhabdus marina (T) FR1087 (DQ781320)
Sphingorhabdus litoris (T) FR1093 (DQ781321)
Parasphingopyxis lamellibrachiae (T) JAMH 0132 (AB524074)
Elkelangia baekryungensis (T) SW-150 (AY608604)
Pacificimonas flava (T) JLT2012 (JX878395)
Sphingomicrobium aestuariivivum (T) AH-M8 (KM591917)
Sphingomicrobium lutaoense (T) CC-TBT-3 (EU564841)
Sphingomicrobium marinum (T) CC-AMZ-30M (JX235672)
Sphingomicrobium flavum (T) CC-AMZ-30N (JX393854)
Aquisediminimonas profunda (T) DS48-3 (KM670029)
Rhizorhapis suberifaciens (T) IFO 15211 (D13737)
Rhizorhapis suberifaciens (T) CA1 (KF437561)
Sphingobium xenophagum (T) BN6 (X94098)
Sphingobium abikonense (T) IAM 12404 (AB021416)
Sphingobium amiense (T) YT (AB047364)
Sphingobium indicum (T) B90A (AY519129)
Allosphingosinicella vermicomposti (T) YC7378 (FJ442859)
Sphingosinicella microcystinivorans (T) Y2 (AB084247)
Sphingosinicella soli (T) KSL-125 (DQ087403)
Stakelama pacifica (T) JLT832 (EU581829)
Stakelama sediminis (T) CJ70 (EU099873)
Hephaestia caeni (T) ERB1-3 (FJ948169)
Allosphingosinicella indica (T) Dd16 (HQ449390)
Sphingomonas echinoides (T) ATCC 14820 (AB021370)
Sphingomonas leidyi (T) ATCC 15260 (AJ227812)
Sphingomonas pituitosa (T) EDIV (AJ243751)
Sphingomonas yabuuchiae (T) GTC868 (AB071955)
Sphingomonas abaci (T) C42 (AJ575817)
Sphingomonas faeni (T) MA-olki (AJ429239)
Sandaracinobacter sibiricus (T) RB16-17 (Y10678)
Sandarakinorhabdus limnophila (T) so42 (AY902680)
Polymorphobacter multimanifer (T) 262-7 (AB649056)
Polymorphobacter glacialis (T) B555-2 (KP013180)
Sphingomonas fennica (T) K101 (AJ009706)
Rhizorhabdus starnbergensis (T) 382 (JN591314)
Rhizorhabdus wittichii (T) DSM 6014 (AB021492)
Rhizorhabdus histidinilytica (T) UM2 (EF530202)
Sphingoaurantiacus polygranulatus (T) MC 3718 (KP274054)
Zymomonas mobilis (T) ATCC10988 (AF281031)
Zymomonas mobilis (T) ATCC29192 (AF281032)
